# Enhancing provider adoption of patient-reported outcome measures (PROMs) through implementation science: insights from two international workshops

**DOI:** 10.1186/s41687-025-00911-3

**Published:** 2025-07-01

**Authors:** Angela C. Wolff, Kate Absolom, Sara Ahmed, Susan J. Bartlett, Maria Jose Santana, Angela M. Stover, Elizabeth J. Austin

**Affiliations:** 1 School of Nursing, Trinity Western University, Langley, BC Canada; 2 Leeds Institute of Health Services Research, Leeds Institute of Medical Research, University of Leeds, Leeds, UK; 3 McGill University, Faculty of Medicine and Health Sciences, Research Institute of McGill University Health Centre, Centre de Recherche Interdisciplinaire en Réadaptation (CRIR), Montreal, QC Canada; 4 McGill University, Faculty of Medicine and Health Sciences, Research Institute of McGill University Health Centre, Arthritis Research Canada, Montreal, Canada; 5 Adjunct Professor Johns Hopkins Medicine, Baltimore, USA; 6 Departments of Paediatrics and Community Health Sciences, Cumming School of Medicine, University of Calgary and Provincial Lead Patient Engagement, Alberta Strategy for Patient Oriented Research Unit, Calgary, AB Canada; 7 Department of Health Policy and Management, Lineberger Comprehensive Cancer Center, UNC-Chapel Hill, Chapel Hill, NC USA; 8 Department of Health Systems and Population Health, School of Public Health, University of Washington, Seattle, WA USA

**Keywords:** Patient-reported outcomes, Routine outcome measures, Patient-reported assessments, Implementation science, Patient-centred care, Implementation

## Abstract

**Background:**

Although the use of patient-reported outcome measures (PROMs) in practice is increasing, successful implementation is contingent on engaging healthcare providers (HCPs). Using Implementation Science (IS), we present the content of two workshops hosted at the International Society for Quality-of-Life annual conferences for individuals seeking to implement PROMs collection and use in their settings. Our goals were to provide workshop participants with knowledge, tools, and resources to prepare HCPs for PROM adoption and to demonstrate tailored strategies to meet context-specific needs.

**Methods:**

An interdisciplinary team with diverse expertise in PROMs implementation delivered two workshops guided by the Capability, Opportunity, Motivation – Behavior (COM-B) model and the Theoretical Domains Framework (TDF). Using dotmocracy, participants were asked to consider, for their local context, the factors most important for changing HCPs’ behaviors to adopt PROMs in daily practice.

**Results:**

The workshops incorporated IS theories, models, and frameworks (TMFs) to identify barriers faced by HCPs, support behavior change, and apply tailored theory-informed implementation strategies to prepare HCPs for PROM integration and evaluate adoption success. The factors rated the most important by workshop participants (*n* = 53) were woven into the discussions to illustrate the most common barriers encountered by HCPs adopting PROMs. Presenters drew on real-world practice and research experiences to identify promising implementation strategies, including education, training, behavioral modeling, persuasion, environmental restructuring, enablement, and audit and feedback to increase the capability, opportunity, and motivation of HCPs.

**Conclusions:**

Given the increasing evidence base supporting the role of PROMs in patient-centered care, it is imperative to understand the mechanisms and best practices for increasing provider adoption of PROMs. This work offers a roadmap for understanding determinants more important to HCPs and systematically selecting theory-informed implementation strategies that may increase the likelihood of HCP adoption of PROMs. Offering tailored HCP training/education programs and implementation strategies can prepare HCPs for timely and effective PROM implementation.

**Supplementary Information:**

The online version contains supplementary material available at 10.1186/s41687-025-00911-3.

## Plain Language summary

Patient-reported outcome measures (PROMs) can make healthcare better in many ways. Yet not all healthcare providers (HCPs) use PROMs, and they sometimes face problems when trying to use PROMs in routine care. We held two workshops that looked at the problems often faced by HCPs and made suggestions to help them adopt and use PROMs on a regular basis. The workshops were guided by Implementation Science, a field that studies how to overcome barriers and improve practice. During the workshops, presenters focused on common issues faced by HCPs, like the lack of resources or support and low knowledge about PROMs. Participants and workshop facilitators then discussed best practices for addressing these issues. The findings from this work can guide future training with HCPs and efforts to increase PROM use in healthcare settings.

**Supplementary Information:**

The online version contains supplementary material available at 10.1186/s41687-025-00911-3.

## Background

The healthcare sector has seen a shift towards patient-centered care (PCC), emphasizing the importance of patients as active participants in their health management. This approach aims to optimize clinical outcomes by focusing on what matters most to patients [1]. Patient-reported outcome measures (PROMs) have emerged as valuable tools to facilitate this by capturing patients’ perspectives on their health, functional status, and quality of life [2–4]. Healthcare providers (HCPs) can use PROMs to guide clinical decision-making, strengthen patient-provider communication, and improve overall care [1, 5–7]. However, implementing PROMs on a wide scale poses significant challenges, as it requires changes to established clinical workflows and provider clinical practice (e.g., role and scope). HCPs must adapt to using these patient-centred assessment tools, which involve integrating PROM responses into care plans and utilizing them for continuous monitoring and decision-making. For successful adoption, PROMs require tailored support, including training for HCPs on what they are and how to incorporate these tools effectively [8–10]. This change often demands behavioral adaptation and systematic planning, as HCPs balance PROMs with other clinical responsibilities. This is where Implementation Science (IS) becomes essential, as it provides structured theories, models, and frameworks (TMF) to understand and address the barriers and facilitators to adopting new practices [11].

Rather than implementation by “trial and error,” IS offers a systematic approach to embedding PROMs into clinical practice by using evidence-based theories and frameworks. This approach is especially important for complex interventions, like PROM use, which involve multiple interdependent components at micro, meso, and macro levels [12]. The Medical Research Council promotes theory use in complex intervention design but offers no clear direction on theory selection or application [12]. IS TMF aids in determining the factors influencing PROM adoption, thereby providing insights into how best to support HCPs. By using IS, healthcare organizations can better design and evaluate the implementation process, ensuring that PROMs are used effectively and sustainably [9]. Despite these benefits, IS-based strategies for promoting PROM adoption are underutilized [9]. Addressing this gap could enhance HCPs’ ability to use PROMs effectively, ultimately benefiting patient care and improving health outcomes.

Given that HCPs work directly with patients at the point of care, the successful routine integration of PROMs in clinical practice is contingent on HCP adoption. Our observations suggest that the implementation of PROMs is frequently led by non-clinicians. Employing IS as a sense-making tool enables research and implementation teams to more effectively understand and address the specific needs of HCPs in this process. This paper illustrates how we applied principles of IS to design and deliver workshops for research and implementation teams seeking to implement PROMs. We highlight the use of IS TMF to understand the needs (e.g., barriers) faced by HCPs, facilitate HCP behaviour change, and prepare HCPs before and during PROM implementation. Finally, we illustrate tailored implementation strategies to meet HCPs’ needs and ways to determine if PROM adoption was successful. For this workshop, we defined HCP as any individual who works directly with patients at the point of care to provide healthcare services (e.g., physicians, nurses, physiotherapists, social workers, etc.).

## Methods

This paper illustrates the application of IS, alongside current evidence on the needs of HCPs, to design workshops that facilitate the adoption of behaviors aimed at integrating PROMs into routine clinical practice. A proposal for a pre-conference workshop was submitted to the International Society for Quality of Life Research (ISOQOL) Annual Conference, a global forum that brings together researchers, clinicians, industry professionals, consultants, and patient research partners. Upon acceptance, an interdisciplinary team of PROM researchers facilitated two workshops (see Table 1). Both workshops were grounded in the same IS TMFs, with the three-hour workshop focusing on the requisite knowledge, skills, and abilities for training/education and the six-hour workshop allowing for greater depth of the same content, along with identifying complementary implementation strategies and evaluation. The workshops were advertised to society members with an intermediate level of knowledge about PROMs implementation. All ISOQOL conference delegates were eligible to register for a fee; however, we anticipated that most participants would be university researchers and implementation teams from healthcare institutions. Before the workshop, participants received an email survey containing closed-ended questions (5 items for the three-hour workshop and 11 items for the six-hour workshop) designed to gather information about their backgrounds, along with one open-ended question to identify questions to be answered. The following sections highlight the underpinnings of the workshop to achieve the desired learning objectives.

**Table 1:** Workshop overview

	Workshop 1	Workshop 2
Year	2022	2023
Length	3 h	6 h
Title	Designing Training and Education to Facilitate Sustained Provider Adoption of Patient-Reported Outcomes in Routine Practice	Making PROMs Work in Practice Settings: Practical Strategies to Support their Implementation by Healthcare Providers
Goal	To provide participants with knowledge, tools, and resources to optimize the development of successful learning activities for PROMs adoption. We will support participants in the planning, development, and delivery of effective PROMs training and education activities for health care professionals.	To provide participants with foundational knowledge, skills, and resources to prepare and motivate HCPs to routinely use PROMs in direct care (i.e., preventive care, chronic care, population-based care). The Consolidated Framework for Implementation Research will guide the workshop to illustrate the intersection between the “user” and “implementation process” domains.
Objectives	1. Apply implementation science theory to characterize the requisite knowledge, skills, and clinical reasoning capabilities of HCPs for both initial and ongoing learning to support PROM use in clinical care. 2. Utilize real-world examples of training / learning across a variety of disease types, patients, and clinician groups. 3. Design training programs and continuing education that focus on the learning needs, capabilities, and motivations of HCPs.	1. Illustrate the knowledge needed to and attitude/beliefs required for optimal application of PROMs to meet the specific needs of clients for preventive care (screening), chronic care (monitory symptoms and functions), and population-based care (interventional care such as oncology and surgery). 2. Demonstrate the skills needed to (a) collect PROMs data (e.g., fill out the PROMs questionnaire), (b) discuss results with patients, (c) score and interpret PROMs, and (d) incorporate PROMs data into clinical reasoning and shared decision making with patients. 3. Evaluate the implementation of PROMs by HCPs (process and outcomes) and identify evidence-based strategies to provide ongoing support for adoption.
Target Audience	An intermediate level of knowledge about PROM in clinical practice and the implementation process.	An intermediate knowledge level about PROMs in clinical practice and the overall implementation process (e.g., selected PROMs and/or implementation experience). Having an implementation case/project to design implementation supports (e.g., training, practice supports, interpretation tools, and decision-making aids) is beneficial.

Note: The number of presenters was 7 in 2022 and 6 in 2023

### Pedagogical considerations

Leveraging principles of adult learning theory, the workshop format included both lecture and interactive activities to allow participants to apply the content to their own context [13, 14]. Workshop presenters had a breadth of experience implementing PROMs in various care settings in the United States, United Kingdom, and Canada and with different members of healthcare teams (e.g., physicians, rehabilitation professionals, nurses, and pharmacists). Real-world practice examples from the presenters were incorporated through the workshops, thereby illustrating the most common barriers encountered by HCPs adopting PROMs and effective implementation strategies [3, 7, 8, 15–20]. The six-hour workshop included a patient partner to share their experiences with HCPs and PROMs. Having more time for the second workshop allowed for interactive activities, enabling participants to apply the content to their local context and engage in discussions to identify promising, theoretically informed implementation strategies to address the prioritized barriers to HCP adoption of PROMs. Before the workshop, registrants were encouraged to read three selected IS articles [11, 21, 22] and several handouts about the IS TMF. Furthermore, we illustrated other resources about PROMs implementation that could be leveraged (see Supplementary Material 1).

### Implementation science approach

**Fig. 1:**
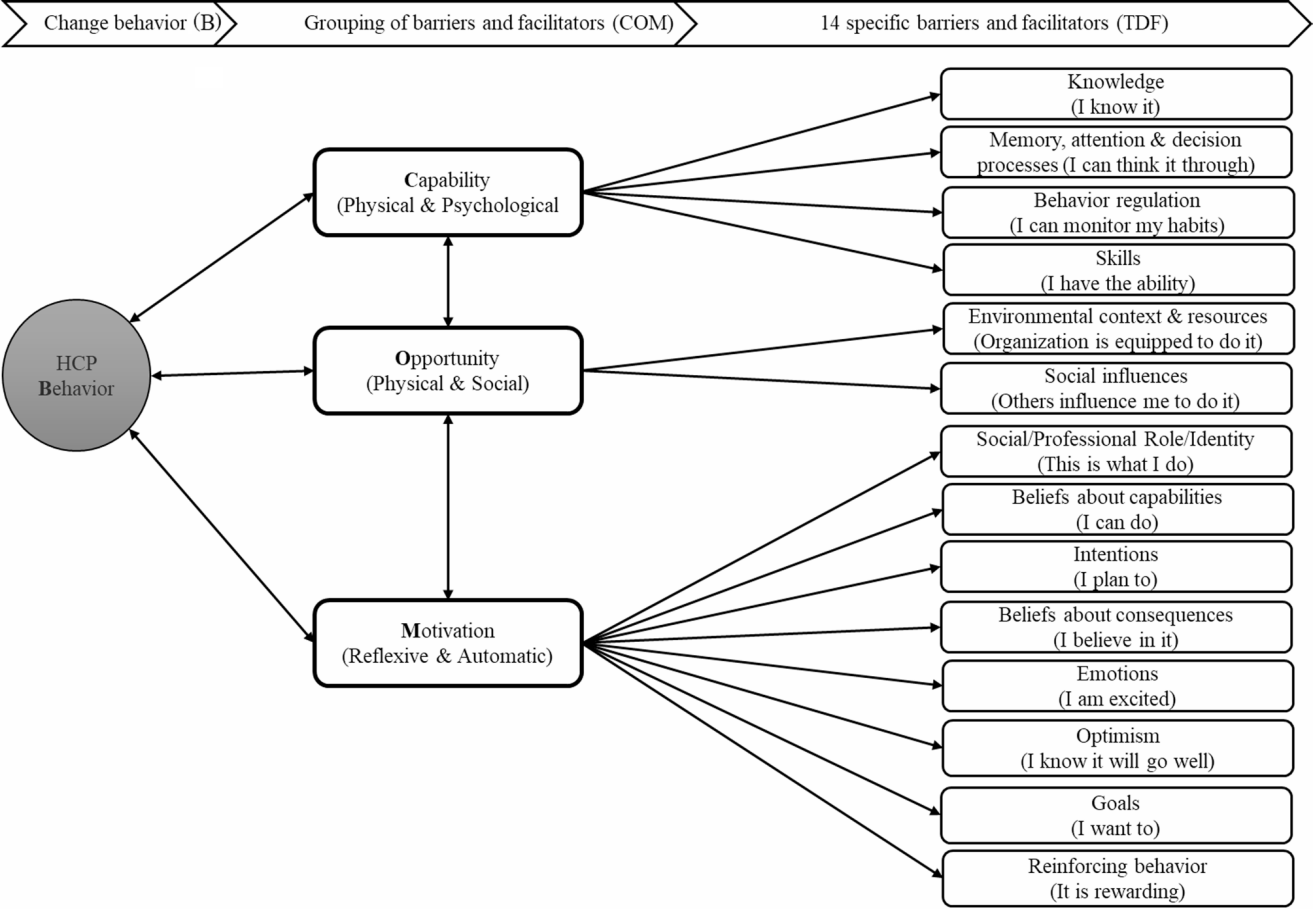
Integration of the Capability, Opportunity, Motivation – Behavior model with the Theoretical Domains Framework. Revised based on [22]

To maintain a focus on the perspectives and needs of HCP stakeholders, workshop content was guided by the Capability, Opportunity, Motivation-Behavior (COM-B) model for understanding individual-level behavior change in the context in which it occurs [21]. COM-B posits that individuals’ capacity to adopt a new behavior is predicated on their capabilities (i.e., physical and psychological), opportunities (i.e., physical and social), and motivation (i.e., reflective and automatic) (see Fig. 1) [21]. To identify the specific needs of HCPs for individual-level adoption, we made use of a more detailed list of 14 domains (determinants), the Theoretical Domains Framework (TDF), to further delineate the COM-B components: (a) capability (knowledge, skills/skill development, decision-making processes, behavioral regulation), (b) opportunity (environmental context/resources and social influences), (c) motivation (social/professional role and identity; beliefs about capabilities; beliefs about consequences, attitudes, reinforcement, intention, goals, emotion) (see Fig. 1) [22, 23].

These complementary frameworks offer useful sense-making tools for comprehensively exploring factors (barriers and facilitators) that influence HCP adoption of PROMs. To overcome barriers and leverage facilitators for a given context, the next step is to systematically select COM-B implementation strategies (i.e., education, training, persuasion, environmental restructuring, incentivization, coercion, restriction modelling, and enablement) [24]. The nine types of implementation strategies (see Table 2) are further broken down into 93 specific strategies, which are likely to be effective in bringing about the desired behavior change [24]. The implementation strategies and evaluation frameworks featured in the workshop were based on the expertise of the presenters and examples from their research [7–9, 15–20].

**Table 2:** Type of COM-B implementation strategies

Type	Definition	Examples
Education	Increasing knowledge or understanding	Providing information (practical and procedural) about PROMs and the specific measure
Persuasion	Using communication to induce positive or negative feelings or stimulate action	Using a patient partner or HCP to share their experience with PROMs to motivate and increase integration
Incentivisation	Creating an expectation of reward	Monitoring (audit) how often the PROM is being completed by HCP
Coercion	Creating an expectation of punishment or cost	Aduit and feedback of HCP using the PROM
Training	Imparting skills	Advanced training for HCP to demonstrate how to administer, scoring, and integrating to inform care
Restriction	Using rules to reduce the opportunity to engage in the target behaviour (or reducing the opportunity to engage in competing behaviours)	Not applicable
Environmental restructuring	Changing the physical or social context	Providing on-screen prompts or cues for HCPs to review the PROM results
Modelling	Providing an example for people to aspire to or imitate	Other HPCs demonstrating the PROM
Enablement	Increasing means/reducing barriers to increase capability or opportunity	Practical support in the clinical setting, adding support in the environment (more computers), plan when to use PROMs

[24]

### Introductory activity

An introductory activity, called dotmocracy [25, 26], was completed with workshop participants after receiving a brief overview of IS TMFs, specifically the COM-B and TDF. Before the workshop, participants were also provided with a two-page handout explaining the 14 domains according to the COM-B components. The goal of the interactive activity was to encourage participants to apply the information to their local context. To that end, participants were asked, “Which factors are most important to change HCPs’ behaviors to adopt patient-reported outcome measurement in daily practice?” Fourteen signs, one for each domain in the TDF, were posted on the room walls. Participants were given five dot stickers and asked to walk around the room to place their stickers on the signs that illustrated the most important factor(s) for HCPs’ adoption of PROMs. Participants could decide how the dots were distributed, for example, they could place all five dots on one sign or place one dot on five separate signs. Once complete, totals for each of the determinants were counted and shared with participants to discuss their impressions.

## Results

Combined, the 53 participants who attended the workshops (see Table 3) were from North America (27%), Canada (21.5%), and Europe (20.5%) and typically affiliated with healthcare institutions (35.5%) or universities (49.5%). About two-thirds of participants had experience implementing PROMs in their settings. The most common types of clinical settings included outpatient clinics (73%), with small numbers in primary care, acute care, and long-term care. Participants implemented PROMs in a wide range of patient populations and health conditions (e.g., oncology, pediatrics, surgery, older adults, maternity, mental health). Within these settings, the most common HCPs implementing the PROMs were physicians (91%), registered nurses and nurse practitioners (86%), and physiotherapists (19%). In the pre-conference survey, participants’ responses to the open-ended question indicated they want to know more about the most effective ways to train HCPs in using PROMs, including flexible formats like videos, one-on-one sessions, and tailored documents. They wanted practical guidance on integrating PROMs into daily clinical workflows while ensuring HCP engagement, buy-in, and sustained use beyond initial funding. There was strong interest in selecting appropriate IS TMF and addressing barriers such as time concerns, relevancy, data usability, and patient participation in remote electronic PROM completion. Finally, participants sought strategies for ongoing training, stakeholder engagement, and actionable use of PROM data to improve communication, clinical decision-making, and patient outcomes.

**Table 3:** Workshop participant demographics

	Workshop 1^1^ count	Workshop 2^2^ count	Average
Number Attended	17	36	26.5
**Affiliation**			
University or research institute	7	21	14
Healthcare institutions (i.e., hospital medical centre, cancer institute, regional health service)	6	13	9
Industry	4	2	3
**Countries**			
United States	4	11	7.5
United Kingdom	4	3	3.5
Europe	4	6	5
Canada	3	9	6
Australia	1	1	1
East Asia	1	6	3.5
**Experience Implementing PROMs**			
Yes	**8**	**17**	12.5
No	4	9	6.5
Unsure	1	3	2
**Patient Population/Health Condition** ^3^			
Oncology/cancer care	1	5	3
Pediatrics	0	4	2
Other (e.g., surgery, ophthalmology, cardiology, older adults, transplant, maternity, mental health, arthritis, multiple)	10	10	10
**Type of Setting** ^3^	(select one)	(all that apply)	
Outpatient	7	13	10
Primary care	1	2	1.5
Acute care	0	4	2
Community	0	1	0.5
Long-term care	0	4	2
Other/multiple	2	1	1.5
**Type of Healthcare Provider Implementing PROMs** ^3^			
Physicians	9	17	13
Registered nurses	4	12	8
Nurse practitioner	4	5	4.5
Physiotherapists	1	5	3
Social worker	1	3	2
Other (general, dietitian, pharmacist, occupational therapist, speech therapist, midwife)	4	5	2.5

^1^Pre-workshop survey sample size = 13

^2^Pre-workshop survey sample size = 29

^3^Responses of those who indicated “yes” to implementing PROMs

**Fig. 2:**
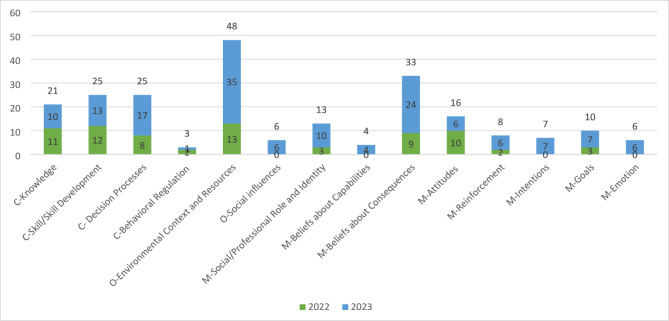
Results from Dotmocracy Prioritizing Activity. Notes: Optimism (not asked). Attitudes are also a component of Beliefs about Consequences (self-efficacy, attitudes, and perceived behavioural control

The results of the dotmocracy activity were used to guide discussion with the participants about the common barriers HCPs encounter when adopting PROMs (see Fig. 2). The most common barriers, although rated differently in each workshop, were: (a) environmental context and resources (opportunity), (b) skills/skills development and knowledge (capabilities), (c) beliefs about consequences (motivation). Furthermore, participants in the first workshop indicated that HCP attitudes (motivation) were a barrier, whereas those in the second workshop reported decision processes (capabilities). Throughout the workshop, emphasis was placed on these barriers. Although presented linearly, we acknowledge that the COM-B barriers are interdependent and synergistic in the real world. For ease of reporting, we will proceed in the order of the acronym COM. Following a discussion of content for each barrier, in the first workshop we emphasized the training needs of HCPs to address these barriers, whereas in the second workshop multiple implementation strategies were suggested, specifically education, training, persuasion, behavioral modeling, environmental restructuring, and enablement [24] (see Table 4).

**Table 4:** Summary of COM-B components, accompanying implementation strategies, and practical examples

Components and Determinants	Questions to Answer^a^	Implementation Strategies^b^	Examples in Practice
**CAPABILITY**			
*Practical knowledge* *(“know what”)* *Procedural knowledge* *(“know how”)* *Skills and skill development*	• What are PROMs? • What do PROMs measure (in general, and for specific care area)? • What are the potential benefits of PROMs to patient care? • How and when are PROMs administered in clinical workflow? • How are PROM scores accessed? • How are PROM scores interpreted? • How do HCPs talk to patients and team members about PROM scores?	**Education** • Conduct local needs assessment to determine knowledge needs (barriers and facilitators) • Develop educational materials • Host educational sessions • Make training dynamic **Persuasion and Modeling** • Identify and prepare champions • Inform local opinion leaders • Identify early adopters	• Distribute brief educational video to provide general knowledge and shape beliefs about PROMs • Share evidence reports about PROMs (e.g., peer-reviewed articles and/or summaries of evidence around PROM use) • Conduct reflection activity where HCPs complete PROM for themselves and discuss reactions to experience • Develop practice aids and/or quick user guides tailored to healthcare providers about PROM score access, interpretation, and communication • Host live demonstration and/or sandbox opportunities to trial PROM use in actual workflow • Develop scripts that offer examples of how to introduce, discuss, and incorporate PROMs into clinical and/or shared decision-making (and strategies of managing challenging scenarios e.g., distressed patients or individuals with multiple complex needs) • Use patient personas and journey mapping to understand patients’ experiences • Develop case studies based on real work-world examples of patient scenarios to demonstrate how PROMs data can add insight on patient health status use of data to direct clinical consultation • Assess HCPs to determine provider needs and tailor strategies
**OPPORTUNITY**			
*Environmental context and resources*	• What challenges do HCP encounter when trying to incorporate PROMs into routine practice? • What would make it easier for HCPs to access and utilize PROM results? • What would make it easier for HCPs to discuss PROM scores during patient consultations?	**Environmental restructuring** • Conduct local consensus discussions • Change physical structure and equipment • Facilitation • Promote adaptability • Assess to determine important barriers and facilitators	• Incorporating templates into clinical workflow that enhance rapid PROM interpretation and response (e.g., templates for responding to PROM scores, incorporating into progress notes or clinical documentation, additional clinical and/or diagnostic criteria) • Placing the PROM on a different color of paper to stand out • Having another clinical team member pre-review the PROM and alert the HCP to a positive score • Creating reminders or prompts to complete PROMs during initial and follow-up patient visits • Conduct an assessment of HCPs to determine provider needs and tailor strategies
**MOTIVATION**			
*Attitudes and beliefs about consequences* *Identity and roles* *Goals and intentions*	• What is the relevance/utility of PROMs for their clinical practice? • Are PROM viewed use as part of their professional role? • What is the goal for adopting PROMs into clinical practice for this setting? • What are the HCPs concerns about using PROMs?	**Persuasion and Modeling** • Assess for readiness and identify barriers and facilitators • Identify and prepare champions • Audit and feedback **Incentivization** • Alter incentive/ allowance structures **Training** • Make training dynamic • Involve both local opinion leaders and early adopters	• Provide educational resources to address negative beliefs (e.g., “Myths and Truths about QOL assessments”) • Incorporate endorsement from locally recognized peers/leaders in training or educational materials e.g., quote or videos of peers describing personal experiences about the importance of PROMs in practice • Communicate the goals of PROMs at the individual level • Use of academic detailing and/or peer-to-peer coaching (e.g., academic detailing) to improve attitudes towards PROM use • Share data about HCP PROM use across the setting • Connect PROM use to performance goals • Facilitate friendly competitions between units and/or teams to incentivize PROM use • Tracking PROM completion rates • Create videos or other resources to illustrate the benefits to patients and HCPs (testimonials) • Conduct an assessment of HCPs to determine provider needs and tailor strategies

^a^ [27]

^b^ [24, 36]

### Component 1: capability

Capability considers an individual’s physical and psychological capability to support the desired behaviour (see Fig. 1). This component is synergistic with both opportunities and motivation in that behaviour change cannot occur without consideration of the interdependent nature of these components [24]. In the introductory activity, important determinants were the knowledge and skills for optimal integration of PROMs by HCPs during client interactions. Knowledge (practical and procedural) is dependent upon learning and developing skills. Practical knowledge provides concrete information for HCPs to understand how PROMs could be incorporated into their current workflow and practice. Procedural knowledge includes information about the procedures and processes to illustrate how PROMs could fit within their existing routines [27]. Table 4 provides an overview of the types of questions that HCPs often want to have answered to understand how PROMs can be used in practice. “General” refers to the necessary foundational knowledge to scaffold the more detailed “specific” knowledge that is distinct to a particular measure, clinical setting, profession, and patient population [27]. Both practical and procedural knowledge are foundational to building the skills, or tacit knowledge, necessary to shift the HCPs’ mental models of practice and mitigate the burden.

Skill acquisition is also an important factor that can promote or hinder PROM integration by HCPs. Specifically, the skills and decision making processes required by HCPs are (a) collecting PROMs data (i.e., having patients complete the PROMs questionnaire); (b) scoring and interpreting PROMs; (b) discussing results with patients, families, caregivers, and other members of the healthcare team; and (d) incorporating PROMs data into clinical reasoning and shared decision making with patients [27]. This illustrates the reciprocal nature of decision-making processes and the knowledge required for integrating the PROMs results into patients’ plan of care. Overall, the goal is to optimize HCPs’ ability and proficiency to embed PROMs into their existing ingrained practices [27].

*Implementation Strategies*. There is a need to provide initial and ongoing education and training support so that HCPs can use PROMs habitually with the least amount of mental energy. To achieve the desired behaviour, education serves to provide foundational knowledge (practical and procedural) while training provides hands-on opportunities to develop the necessary cognitive, physical, and communication skills required to act on PROM scores for clinical decision-making and care planning. While necessary, education and training are insufficient for behavior change. Coupled with persuasion and modelling from influential persons working with HCPs (e.g., champions, local opinion leaders, and early adopters) allows for opportunities to have trusted and credible colleagues enact the proposed behaviour change (e.g., PROMs administration and scoring) and provide feedback (see Table 4). This just-in-time support allows a safe learning environment for HCPs to imitate and receive feedback while adopting the intended behaviour in their real-world practice. This enablement, or support, is critical to reinforce the desired behaviour. Furthermore, other capabilities interconnected with physical and social environment barriers (i.e., clinical workflows, physical resources, electronic resources, people) in the local context can be addressed. For example, one strategy is to develop an accessible user guide—distinct from the PROM developers’ manual—for HCPs, incorporating communication scripts for PROM administration and demonstrating its relevance to clinical decision-making and care planning. Finally, more advanced skill acquisition would be to integrate PROM results into a shared decision-making approach [28]. Other examples of implementation strategies for capabilities are illustrated in Table 4.

### Component 2: opportunity

Opportunity considers the factors directly impacting end-users, not only at the micro but also organizational level, that encourage or discourage the adoption of behavior, often a reflection of the physical or social environment (see Fig. 1) [24]. Therefore, it was not surprising that workshop participants identified the construct of environmental context and resources as the most important and common determinant. The discussion further clarified that when context and resources are attended to, resources are a facilitator for provider adoption; yet, when overlooked can become a barrier to the uptake of PROMs in routine practice. Participants identified examples of limited electronic health record integration, lack of adequate staffing, and time constraints resulting from busy outpatient care settings. Yet, the discussion also acknowledged that the construct of environmental context and resources is quite broad, making it difficult to translate implementation experiences from setting to setting. To address this, workshop organizers defined four critical aspects of the environment that are known to influence provider adoption of new behaviors, including PROMs: (a) clinical workflows; (b) physical resources; (c) electronic resources; and (d) people (e.g., clinical and administrative staff).

*Implementation Strategies.* To address barriers stemming from environmental context and resources, workshop presenters illustrated real-world examples of strategies to facilitate environmental restructuring and enablement (see Table 4) to reduce physical and social contextual factors that make adoption more difficult. Environmental restructuring may address several or all dimensions of the physical and social environment (i.e., clinical workflows, physical resources, electronic resources, and people), as appropriate for the local context [17, 24, 29]. For example, when HCPs lack the time needed to review PROM scores during a clinic visit, implementation teams might consider integrating PROM scores directly into the electronic clinical workflow (i.e., reducing the need for additional clicks and/or time spent in the electronic health record) and visually presenting scores in ways that enable rapid review (i.e., reducing cognitive load for interpretation) [29, 30]. Table 4 includes additional examples of environmental structuring strategies specific to PROM adoption.

### Component 3: motivation

Motivation considers internal decision-making to engage in the desired behaviour (i.e., use PROMs), often influenced by internal and external factors (see Fig. 1) [24]. Workshop participants identified attitudes and beliefs about consequences as mattering most for provider adoption of PROMs. Positive initial experiences impact HCPs’ future intentions to continue using patient-reported assessment tools. Conversely, motivational barriers are encountered when negative views or experiences occur [27]. The most common attitudes and beliefs about PROMs have focused on the benefits outweighing the burdens to both HCPs and patients in four main areas: (a) tools and results are clinically relevant or meaningful; (b) practical way to facilitate communication; (c) beneficial to the patient; and (d) performs well psychometrically (reliability, validity and sensitivity to change) [27, 31, 32]. Collectively, the beliefs and attitudes are weighed against one another in consideration of whether to adopt PROMs into practice. For example, adoption is less likely when HCPs have negative attitudes about the value of PROMs, including perceptions of low clinical utility and/or patient benefit, limited accuracy, or difficulty accessing or using the PROMS. HCPs may also view PROMs as a burden to use or not concordant with a patient’s clinical status. Conversely, when HCPs embrace PROMs to start a conversation, they are more likely to recognize how they can build rapport and improve communication between HCPs and patients [27].

HCPs also may have discordant beliefs about how PROMs align with their identity and role. HCPs may consider the degree to which they view PROMs as part of their clinical role and responsibility and how widely PROMs are used among other HCPs like them [27]. Finally, motivation may vary depending on the degree of clarity about the goals and intentions for PROM use. For example, when the goal for incorporating PROMs into clinical practice is unclear, HCPs may be less likely to adopt them into practice. HCPs often express fears attributed to both internal and organizational expectations as well as patient expectations. This can translate into perceived resistance to changing their practice [27]. The motivational component is also intertwined with capability and opportunity, such that low knowledge about how to use PROMs and perceived barriers to using them routinely will also decrease motivation for adoption. Synergy across the three components is necessary for successful and sustained adoption.

*Implementation Strategies*. Two effective social psychology strategies that can increase motivation to adopt PROMs include persuasion and modeling [24]. In the context of PROM adoption, these can encompass several activities (see Table 4). Negative beliefs and misconceptions about PROM utility and reliability can be addressed through education. Negative attitudes about role identity and the value of PROMs for clinical practice can be targeted with peer role modeling (i.e., local clinician champions), patient testimonies, or consensus discussions. Clarifying goals for PROM use is essential. Importantly, goals should be developed collaboratively with HCPs and reflect intended impacts on care quality – including shared decision-making, patient-provider communication, and patient engagement – not just quality improvement. In some cases, using positive or negative incentives may also increase motivation, especially in scenarios where adoption stalls or low adoption persists [33].

### Component 4: behavior

The final component of COM-B is the execution of the behavior itself, which may encompass adoption (routine use), fidelity (as intended), and sustainment (ongoing execution over time and through changing circumstances) [24]. By providing education, training, and other implementation support that holistically identifies and addresses the capability, opportunity, and motivation determinants for HCPs to use PROMs in routine practice. However, the implementation of any behaviour change is dynamic and may wax and wane in response to changing internal and external factors. As a result, implementation teams should consider: (a) defining and measuring core competencies of provider education/training that need to be maintained; and (b) engaging in ongoing monitoring and evaluation of provider use of PROM.

*Core competencies for maintaining provider adoption of PROM*. While initial efforts to prepare HCPs to use PROMs may be multifaceted and intense, over time a smaller set of competencies need to be maintained and/or refreshed to sustain PROM use. For example, while knowledge and skill development about how to interpret a PROM may not need to be repeated, procedural knowledge about how to integrate them into shared decision-making may benefit from refresher support. Implementation teams should also consider training needs for new staff, since approaches to training delivery may differ (i.e., 1:1 training for onboarding a new staff person versus group training that might have occurred initially).

*Ongoing monitoring and evaluation of provider use of PROM.* Evaluation of provider use over time can offer new insights into training effectiveness, learning gaps, and tailoring of training strategies. Implementation evaluation frameworks, such as the Reach, Effectiveness, Adoption, Implementation, and Maintenance (RE-AIM), can offer a structure for evaluation plans [34]. For example, education and training evaluations might assess reach (e.g., number of eligible HCPs involved in training) and effectiveness (e.g., the impact of training on increased knowledge, skills, and beliefs) immediately following training completion as well as over time to identify potential drift and/or emerging barriers to use.

## Discussion

Two workshops were developed and delivered to diverse attendees (*n* = 53) at consecutive annual ISOQOL conferences to illustrate the application of IS to focus on the needs of HCPs as end-users during PROM implementation. Through our introductory activity (dotmocracy), participants identified relevant domains and deterministic factors most likely to influence HCPs adoption in their settings. In alignment with the original workshop design, these common barriers were paired with possible implementation strategies that would enhance HCP knowledge base, foster skill acquisition and decision-making processes about PROMs; positively shape attitudes and beliefs; and modify the environmental context and resources.

Prior research has identified barriers to HCPs’ buy-in when integrating PROM into routine practice [2, 8, 29, 32]. Although some researchers have evaluated evidence-based approaches to increase HCP adoption, the extent to which IS TMF underpin these efforts is unclear. This represents a significant missed opportunity to utilize effective evidence-based implementation strategies specifically matched to the needs of HCPs, thereby resulting in changed behavior. We bridged IS and practice-based experience to systematically identify promising approaches to address common barriers encountered to thereby increasing HCP adoption of PROMs in routine practice. Through a multi-year collaboration among researchers involved in PROM implementation and two international workshops, the process modeling presented in this workshop provides a roadmap for future research and implementation teams seeking to identify, understand, and effectively address determinants of PROM adoption among HCP groups, to select tailored implementation strategies that facilitate clinician behavior change. Central to this will be pairing IS TMF with the specific experiences and needs of local implementation contexts, as the specific application of IS will vary context to context.

Central to understanding provider adoption of PROMs is unpacking the constructs that contribute to the behavior change of HCPs, including the COM-B components and TDF determinants. Prior work has described barriers related to HCP knowledge about PROMs. For example, Frederickson et al. (2016) found that half of HCPs did not know how to interpret the clinical meaning of PROM scores routinely gathered in their primary care setting [35]. Yet knowledge alone is inadequate, and without efforts to increase practical and tacit knowledge may be imprecise and ineffective. By leveraging IS, we were able to clarify that knowledge can include dimensions of practical knowledge, procedural knowledge coupled with skill development to shape the decision-making processes of HCPs. By leveraging our multidisciplinary experience, we characterized core knowledge and skills for PROM use, including activities of delivering, accessing, reviewing/interpreting, and discussing PROM scores with patients and other stakeholders. This level of specificity can greatly aid the design of implementation supports aiming to increase provider knowledge, helping to transform the strategy of “training” – a blunt instrument on its own – to a strategy with increased effectiveness and replicability. Similarly, Ahmed et al. (2021) and Eilayyan et al. (2020) study, have assessed the needs of HCPs before PROMs implementation to match strategies to invest time and resources on areas that matter the most to end-users [16, 20].

To understand what matters most to HCPs, we coupled our expertise with participants’ insights from the introductory dotmocracy activity. This approach – pairing theory with lived experience in the local context – can help future research and implementation teams focus their efforts on participant needs when designing education, training, and other implementation strategies to increase provider adoption. Further, we acknowledge that these components are interrelated, synergistic, and often have a compounding impact on provider adoption. For example, a lack of procedural knowledge about how to access PROM scores and/or poor skills to interpret scores, combined with environmental barriers such as poor integration in the electronic workflow, will in tandem decrease motivation to use PROMs routinely. While prior research echoes the importance of these factors, little research has explored and explained the behavioral pathways and mechanisms that lead to adoption. This work provides a first step towards a common understanding of the PROM adoption process and a foundation for future research on promising implementation strategies. Although no one size fits all in the workshop, we illustrated how various determinants and end-user needs were met during various implementation projects. Depending on the stage of the project, once the initial needs of HCPs are met, there may be a need to address deeper psychological aspects of changing behaviour (e.g., social influence, beliefs about capabilities, intentions, and behavioral regulation). While not initially rated a concern by workshop participants in the dotmocracy activity, HCPs’ agency, self-efficacy, and psychological desires can be important for behaviour adoption [24].

There are several limitations to acknowledge in this work. First, while the workshop presenters and participants represented different countries, research and practice settings, and clinical areas, they do not reflect all experiences of PROM use in clinical practice. Second, participants were members of the ISOQOL who might support HCP adoption of PROMs, and it was not feasible to obtain information about the extent of their involvement with and knowledge about PROMs implementation or whether they were HCPs themselves. We relied on our research expertise in PROMs implementation to design the workshop and assumed that participants would come with diverse backgrounds and levels of expertise. Finally, although an intermediate level of knowledge about PROMs implementation was recommended, workshop attendees had varying levels of knowledge and experience with PROM implementation and IS TMF, which likely influenced the workshop activities and discussion. This workshop and the learnings presented in this paper are intended to reflect an example of how IS – the COM-B model and TDF – can be used to systematically appraise and identify implementation needs, but future work is needed to continue to evaluate what implementation approaches work best for different PROM implementation contexts.

## Conclusions

HCP adoption of PROMs is an essential but understudied component of advancing PROM use in routine care. Given the increasing evidence base supporting the role of PROMs in catalyzing patient-centered care, efforts to understand mechanisms and best practices for increasing HCP adoption of PROMs are imperative. Critical to effective implementation, which is complex and dynamic, is consideration of the individual-level determinants that contribute to adopting PROMs. As a result, these ISOQOL workshops were anchored in IS, specifically the COM-B model and TDF, and practice-based experiences [3, 7, 8, 15–20]. Using the COM-B components and TDF determinants offers a roadmap to help identify and bolster potential facilitators while mitigating various barriers and uncovering potential blind spots necessary for selecting tailored, theory-informed implementation strategies. This work can guide future research and implementation teams seeking to identify implementation strategies to change ingrained HCP behaviour patterns, thereby increasing the likelihood of HCP adoption. Using a theoretical approach allows for efficient and effective use of resources for PROM implementation projects to focus beyond the needs of the organization, but also the end-users.

## Electronic supplementary material

Below is the link to the electronic supplementary material.

Supplementary Material 1

## Data Availability

Not applicable.

## Abbreviations

COM-B: Capability, Opportunity, Motivation-Behavior model
HCP: Healthcare provider
IS: Implementation science
ISOQOL: International Society for Quality of Life
PCC: Patient-centred care
PROM: Patient-reported outcome measure
TDF: Theoretical Domains Framework
TMFs: Theories, models, and frameworks
