# Supplementary material for: Enhancing provider adoption of patient-reported outcome measures (PROMs) through implementation science: insights from two international workshops
Source: J Patient Rep Outcomes. 2025 Jul 1;9:77. doi: 10.1186/s41687-025-00911-3 (PMC12214070; doi:10.1186/s41687-025-00911-3)
Supplement: Supplementary file 1 — Supplementary Material 1 [file 41687_2025_911_MOESM1_ESM.docx]

Supplementary Material 1. External resources to further aid the design of HCP education and training

| **Resource Name** | **Citation and/or weblink** |
| --- | --- |
| **ISOQOL Users Guides** | International Society for Quality of Life Research (prepared by Aaronson N, Elliott T, Greenhalgh J, Halyard M, Hess R, Miller D, Reeve B, Santana M, Snyder C) (2015) User’s Guide to Implementing Patient-Reported Outcomes Assessment in Clinical Practice.  International Society for Quality of Life Research (prepared by Chan E, Edwards T, Haywood K, Mikles S, Newton L) (2018). Companion Guide to Implementing Patient Reported Outcomes Assessment in Clinical Practice.  Both available at: [(isoqol.org)](https://www.isoqol.org/wp-content/uploads/2019/09/2015UsersGuide-Version2.pdf) |
| **PROTEUS Consortium Practice Guide** | Crossnohere NL, Anderson N, Baumhauer J, et al (2024) A framework for implementing patient-reported outcomes in clinical care: the PROTEUS-practice guide. Nat Med 30:1519–1520. https://doi.org/10.1038/s41591-024-02909-8  Available at: <https://theproteusconsortium.org/> |
| **ePROs in Clinical Care** | LeRouge C, Austin E, Lee J, et al (2020) ePROs in Clinical Care: Guidelines and Tools for Health Systems. Seattle, WA: CERTAIN, University of Washington.  Available at: https://becertain.org/epros |
| **PROmunication tool** | Skovlund PC, Ravn S, Seibaek L, et al (2020) The development of PROmunication: a training-tool for clinicians using patient-reported outcomes to promote patient-centred communication in clinical cancer settings. J Patient Rep Outcomes 4:10. https://doi.org/10.1186/s41687-020-0174-6 |
| **Patient-Centered Outcomes Research at Johns Hopkins** | Video  <https://www.youtube.com/watch?v=zUFBJMXKTv8> |
| **Resources for Patients and HCPs about Quality of Life** | Learn about Equitable People-centred Health Measurement (see Applications in Healthcare)  Quality of Life Assessment Support Team (2024)  [www.healthyqol.com/older-adults](http://www.healthyqol.com/older-adults)  <https://www.youtube.com/watch?v=TDL5C9SEBvo&t=5s> |
| Note: This is not intended to be a comprehensive list and access to resources may change over time | |
